# Prognostic Factor and Serum Biomarker Changes in Non-Surgical Hepatocellular Carcinoma Patients Treated with Radioembolization

**DOI:** 10.47829/jjgh.2025.11103

**Published:** 2026-02-12

**Authors:** BI Carr, HG Gozukara Bag, R Kutlu, E Karabulut, V Ince, S Yilmaz

**Affiliations:** 1 Liver Transplant Institute, Inonu University Faculty of Medicine, 44280, Malatya, Turkey; 2 Department of Biostatistics, Inonu University Faculty of Medicine, 44280, Malatya, Turkey; 3 Department of Radiology and Liver Transplant Institute, Inonu University Faculty of Medicine, 44280, Malatya, Turkey

**Keywords:** HCC, Radioembolization, Prognosis

## Abstract

**Background:**

Transarterial radioembolization (TARE) with 90Y is a primary therapy for hepatocellular carcinoma (HCC), including patients with portal vein thrombosis (PVT). Patient survival subgroups post TARE have not been clearly defined.

**Aims:**

To identify prognostic factors in patients treated with TARE.

**Methods:**

Non-surgical HCC patients (n=116) were treated with TARE and prospectively followed for survival and changes in blood parameters relating to inflammation and tumor indices.

**Results:**

Baseline parameters with the highest hazard ratios (HR>2) for survival were C-reactive protein, alpha-fetoprotein (AFP), maximum tumor diameter (MTD) and PVT. A 2-parameter prognostic model was developed, consisting of serum AFP and C-reactive protein, which was especially significant in patients with PVT. Temporal changes post TARE in ALBI and serum gamma glutamyl transferase (GGT) levels occurred in patients with both high and low serum AFP. A network phenotyping strategy (NPS) showed that patient phenotypes also change after TARE. Post TARE reduction in AFP or GGT or increase in albumin levels were associated with improved survival.

**Conclusions:**

Changes in serum CRP, AFP, GGT, albumin, ALBI, and NPS phenotype occurred early after TARE. Changes in AFP, GGT and albumin related to survival and may be useful in selecting patients for additional treatment.

## 2. Introduction

Transarterial radioembolization with (TARE) Yttrium-90 (90Y) has increasingly become part of the therapeutic armamentarium for locoregional therapy for patients with non-metastatic hepatocellular carcinoma (HCC) in recent years, owing to its effectiveness, relatively low toxicity [1–3] and comparable or better response and survival rates to transarterial chemoembolization (TACE) [3–5]. It is also relatively safe compared with TACE in patients with portal vein thrombosis (PVT) [6,7]. Despite the burgeoning literature on its use, precise knowledge is still lacking concerning its mechanisms, predictors of response and resistance, optimal uses and combinations with other treatment modalities. In countries such as Turkiye with a developing economy, many patients are too poor for repetitive transport to tertiary medical centers such as ours and the availability of cheap and useful HCC biomarkers would be beneficial in their treatment follow-up. In this paper, we have explored several potential seromarkers, especially for the large proportion of HCC patients with low baseline levels of alpha-fetoprotein (AFP).

## 3. Methods

One hundred and sixteen patients with unresectable and untransplantable hepatocellular carcinoma (HCC) were treated with TARE and followed for changes in serum HCC and inflammation parameters.

Demographic and clinical characteristics of the HCC patients analyzed for this study were: age, gender, baseline and last alpha-fetoprotein (AFP), liver function and complete blood count values, maximal baseline tumor diameter, and presence or absence of macroscopic portal vein invasion by tumor (PVT) on the CAT scan. ALBI grade was calculated by use of: www.mdcalc.com ALBI grade for HCC. NPS phenotype was calculated by use of the NPS web tool: https://apkatos.github.io/webpage_nps.

A diagnosis of HCC was established by percutaneous biopsy or noninvasively based on presence of a hepatic mass greater than 2 cm diameter with characteristic imaging findings in the setting of liver cirrhosis as per EASL guidelines. Treatment was performed on an inpatient basis, and patients were typically not discharged home for 24 hr after the procedure for post-TARE safety observations.

Inclusion criteria included patients with HCC who were considered unamenable to surgical treatment, age >18 years, absence of metastases, lung shunt <20%, prior informed consent for the radioembolization procedure and treatment; ECOG performance status <3 and adequate hematologic, renal and liver function tests for a safe interventional procedure.

## 4. Radioembolization Procedures

Patients were treated as per published guidelines (8–10) and underwent pre-treatment angiographic mapping of the abdominal aorta and hepatic arteries. Planar scans of the lung and liver area in anterior and posterior views were acquired after injection of 99mTc-labelled albumin macroaggregated albumin (99mTc-MAA) into selected arterial branches followed by SPECT (until 2006) or SPECT/CT scans. Radioembolization was delivered using Yttrium-90 resin microspheres (SIR-Spheres; Sirtex Medical Limited, North Sydney, NSW, Australia) or glass microspheres (Therasphere, Boston Scientific, Boston MA, USA). Dose calculation performed through personalized dosimetry planning, considering optimal absorbed doses by tumoral and non-tumoral volumes, was used by the treating interventional radiologist.

## 5. Statistical Methods

The Shapiro-Wilk test was used to assess the normality of the quantitative data. Comparisons between two independent groups were performed by the Mann-Whitney U test. Median, interquartile range, minimum, and maximum values were used to **summarize** the quantitative data. Distribution of the qualitative data was presented by count and percentage, and the continuity-corrected chi-square test was applied for comparisons. The Kaplan-Meier method was used for survival estimations, and the Log-Rank test was used for survival comparisons between groups. Univariate Cox regression analysis was used to obtain the Hazard Ratios. In all analyses, the two-sided significance level was considered as <0.05. IBM SPSS Statistics for Windows, Version 26.0 (Armonk, NY: IBM Corp.) was used for statistical analyses.

The study protocol was approved by the ethical committee of the Inonu University institutional review board (IRB) for non-interventional studies (2025/8125) for data collection and analysis, and conducted in adherence to the Declaration of Helsinki. Since this was a non-intervention retrospective study, patient informed consent was not required for the study evaluation, although each patient signed an informed consent for the procedure.

## 6. Results

### 6.1. Survival in The Total Cohort and in Parameter Subgroups

Survival in this TARE-treated patient cohort was calculated by Kaplan-Meier analysis (Table 1a). Mean survival was 19.95 +1.59 months and median survival was 13.93 + 3.24 months. We and others have previously reported that HCC prognosis depends on both tumor and liver factors, especially liver inflammation [11–12]. Accordingly, these factors were dichotomized as reported previously [13–15] and examined for their relationship to survival (Table 1b). The dichotomized parameters with the highest hazard ratios were, in descending order, C-reactive protein or CRP (HR 2.787), maximum tumor diamter or MTD (HR 2.512), alpha-fetoprotein or AFP (HR 2.149), portal vein thrombosis or PVT (HR 2.032), ALBI (HR 1.978) and albumin (HR 1.857). For each of these dichotomized parameter pairs, survival was 3–4 fold different.

### 6.2. A Model for Survival Using Parameter Combinations.

A model was constructed for combinations of the single parameters with highest HRs (Table 2). The patient combination in the total cohort of CRP≤1 & MTD≤5cm & AFP<100 (low CRP plus low MTD plus low AFP) had a median survival of 32.03 months, whereas the combination of CRP>1 & MTD>5cm & AFP≥100 (high CRP plus high MTD plus high AFP) had a median survival of 3.83 +1.68 months (log rank p<0.001) and HR 10.3. Using only the 2 parameters of CRP and MTD, survival differences were also significantly different, with an HR of 4.3. In the clinically important group of poor prognosis patients with PVT, the 2 parameter combination of CRP plus AFP also was significantly associated with 2 prognostic groups, with median survival of 22.70±2.71 and 4.27±0.54 months respectively, for patients who had low serum CRP plus low AFP values versus high serum CRP plus high AFP values, log rank p<0.001 and HR of 8.658. This is potentially useful in selecting patients with PVT who might benefit from future definitive HCC surgery or other therapies.

### 6.3. Time-Dependent Serum Parameter Changes Post TARE

**Figure 1:**
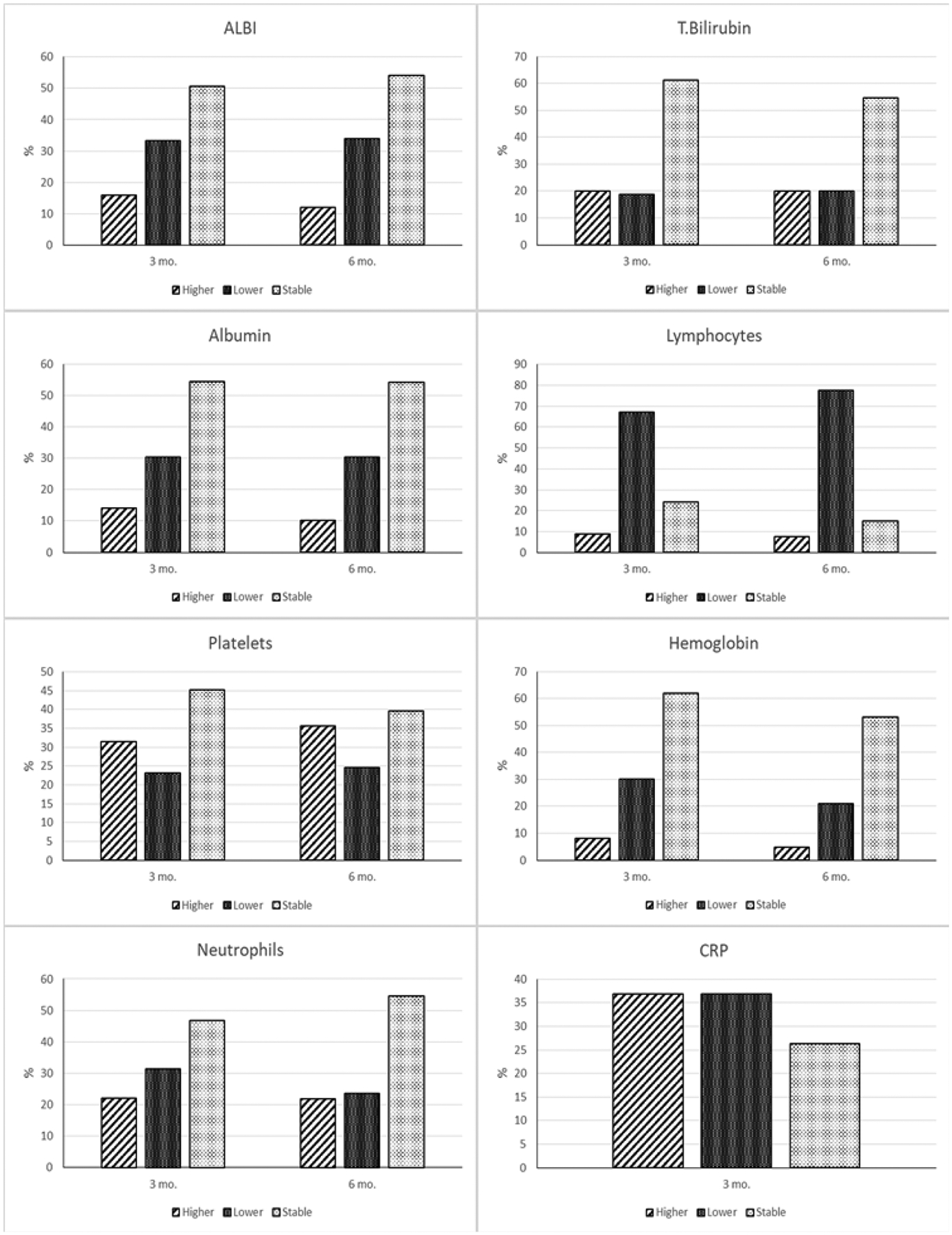
Serum parameter changes with time post TARE.

Figure 1 shows changes in several parameters at 3 and 6 months post TARE. For the prognostically important ALBI score, 33.9% of patients had improved (lower) scores, 12.6% had worse scores (higher), while 53.4% of patients had unchanged scores at 3 months post TARE, compared with baseline levels. The findings were similar at 6 months. For total serum bilirubin, 20% of patients had higher (worse) serum levels, 18.6% had lower (improved) serum levels and 61.3% had stable serum levels at 3 months, compared with baseline. The changes at 6 months post TARE were similar. For serum albumin, 14.2% of patients had higher than baseline levels (improved), 30.3% of patients had lower than baseline levels (worsened) and 54.4% of patients had stable levels at 3 months, compared with baseline levels. Again, the results at 6 months post TARE were similar. Blood platelet levels were higher (impoved) compared with baseline values in 31.5% of patients, lower (worse) compared with baseline values in 23.2% of patients and unchanged in 45.3% of patients at 3 months post TARE. There were nearly identical findings at 6 months post TARE. For blood CRP levels, 36.8% of patients had higher (worse) values at 3 months compared with baseline, 36.8% of patients had lower (improved) values compared with baseline and 26.3% of patients had stable values. There were insufficient 6 month survivors with CRP data.

### 6.4. Changes in ALBI, AFP, GGT, Albumin and NPS Phenotype Post TARE

Owing to the importance of the ALBI score in HCC prognostication [16,17], ALBI changes were examined separately in patients with high (>100 IU/mL) or low serum AFP levels (Table 3 [A1] and [A2]. For patients who had high serum AFP levels, ALBI was decreased from grade 3 to 2 or 1, or from grade 2 to 1 (improved) in 32.7% of patients had a decrease (improved), increased from grade 1 or 2 (worsened) in 12.7% of patients and stable in 54.5 % of patients at 3 months post TARE. Very similar findings were seen in patients with low serum AFP levels, showing that the ALBI score is useful in HCC patient groups with both high and low serum AFP levels.

AFP measurements have been found useful in monitoring responses in HCC patients who were treated with TARE and the levels have been reported to correlate well with tumor measurements]^18^,^19^]. AFP levels at 3 months post TARE in our cohort were decreased (improved) in 67.3% of those patients who had elevated baseline AFP levels, increased (worsened) in 21.8% of the patients who had elevated baseline AFP levels and were stable in 10.9% of patients with elevated baseline AFP levels, as shown in Table 3B (i). The responses in AFP levels were associated with changes in survival, as shown in Table 3B (ii), with a 50% decrease in AFP level being associated with 34.03±3.17 months survival, whereas a decrease of <20% or an increase was associated with a significantly different mean 10.17±2.58 months survival.

However, AFP cannot be used as a serum biomarker in that substantial percentage of HCC patients who have normal serum AFP levels at baseline [20,21]. For these patients with low baseline serum AFP levels, it has previously been shown that changes in serum GGT levels can be a useful biomarker [22]. GGT changes were found at 3 months in the patients of this cohort with low baseline AFP levels. In patients in this cohort with low baseline serum AFP levels, 34.9% of patients had a decrease in GGT levels (improved) at 3 months post TARE compared with baseline, 48.8% of patients had an increase (worsened) in GGT levels at 3 months post TARE compared with baseline and 16.3% of patients had stable GGT levels, as shown in Table 3C (i). Similar GGT changes were found in patients with high serum AFP levels. Patients who had a decrease or stable serum GGT levels at 3 months post TARE had a median survival of 22.7 months, compared to 10.9±5.85 months in patients who had an increase in GGT levels. However, the result did not quite reach significance, p=0.063, as shown in Table 3C (ii).

Changes in ALBI in relation to survival could not be calculated, due to the small patient numbers with high ALBI grades. However, we found changes in serum albumin levels that significantly related to survival (Table 3D). Patients who had an increased albumin at 3 months post TARE had a mean survival of 34.76+4.04 months, compared to a mean survival of 31.59+2.56 months in patients with stable albumin, and compared to a mean survival of 16.38+2.74 months in patients with a decreased albumin, p<0.001. The network phenotype strategy (NPS) was developed to integrate 17 standard clinical parameters into 25 prognostically-related HCC phenotypes, 1–25 with phenotype 25 being the most aggressive with the worst prognosis and phenotype 1 having the best prognosis (23, 24). NPS was used here for the first time to evaluate changes of HCC phenotype over time, as observed after TARE therapy (Tables 3E). We found that 27.3% of patients had a decrease in phenotype (less aggressive), 40.9% of patients had an increase in phenotype (more aggressive) and 31.8% of patients had stable phenotype, as shown in Table 3E (i). There was a wide range in phenotype response, depicted in 8 patients who started with the highest baseline phenotype, as shown in Table 3E (ii).

### 6.5. Clinical Characteristics of the Dichotomized Subgroups

The clinical characteristics of the dichotomised subgroups for the 4 parameters with highest HR values, namely CRP, AFP, MTD and PVT are shown in Table 4. The 2 CRP subgroups (Tables 4a and 4b) were significantly different with respect to MTD, AFP, percent of patients with PVT, tumor focality, serum albumin and levels of gamma glutamyl transferase (GGT). The 2 AFP subgroups (Tables 4c and 4d) significantly differed only for MTD, platelets and neutrophils. The 2 MTD subgroups (Tables 4e and 4f) were significantly different with respect to AFP, percent of patients with PVT, tumor focality, GGT, ALKP, AST, platelets and neutrophils. The 2 PVT subgroups (Tables 4g and 4h) were significantly different with respect to MTD, AFP, tumor focality, total bilirubin, CRP, GGT, ALKP, AST, platelets and neutrophils. Thus, each dichotomized pair had a slightly different pattern of clinical characteristics. Interestingly, only the CRP pair was significantly different for all 4 tumor characteristics that were studied, namely MTD, AFP, PVT and tumor focality. Total serum bilirubin was significantly different only in the PVT dichotomized pairs and the AFP dichotomized pairs had significant differences only for MTD, platelets and neutrophils.

## 7. Discussion

The median survival (Table 1a) of 13.93±3.24 months for the total cohort (mean 19.53±1.59 months) is similar to survival reported by others and better than survival recently reported for randomized trials involving sorafenib (25, 26). Using several parameters used in other HCC studies and their cutoffs (14, 15), we found here that there were significant survival differences using tumor factors (AFP, MTD and PVT) and inflammation factors (albumin, ALBI and CRP) as shown in Table 1b.

A model was constructed for combinations of the parameters associated with the highest HR. The combination of low CRP, AFP and MTD values was associated with significantly much longer survival (median 32.03 months) than the combination of high CRP, AFP and MTD values (median 3.83+ 1.68 months), for an HR of 10.3. Just as interesting, was the parameter combination for PVT positive patients known to have poor prognosis. Using only baseline serum CRP or AFP levels (there were not enough PVT patients to analyze the triple parameter combination) there were significant survival differences in PVT positive patients who had low CRP or AFP levels, compared to patients with high levels. The high CRP plus high AFP combination patients had an HR of 8.658 compared to the low CRP plus AFP combination (Table 2) for these PVT positive patients. This identifies a subgroup of otherwise poor prognosis (PVT positive) HCC patients who had a 5-fold increase in survival compared with other PVT positive patients, providing a possible selection criterion for subsequent liver transplantation or even other therapies.

Many of our patients are poor and live at a distance from a tertiary center such as ours, so that it can be difficult for them to return for follow up scans, or sometimes even to geographically close laboratory follow up tests, once they learn that definitive surgical therapy is not being offered. It would thus be important for their follow up if simple and cheap laboratory tests could be shown to facilitate their post-TARE management and decision-making. For this reason, we were interested to examine non-radiological, laboratory test options. We examined several, including albumin (Fig 1), ALBI, AFP, GGT and a network phenotyping strategy (NPS) as shown in Fig 1 and Table 3. Baseline patient ALBI has been previously shown to be prognostically useful in HCC patients (16, 17, 27, 28, 29). We found that ALBI was similarly useful both in patients with elevated and in patients with low serum AFP levels (Table 3A). This is clinically useful, as there are currently no other validated HCC markers for patients without abnormal (high) AFP levels. This is similarly true for GGT measurements and responses in patients with low serum AFP levels (Table 3C) as reported in other HCC series treated without TARE (30, 31). Our patients who had either baseline elevated or normal serum AFP levels, had similar GGT responses, as shown in Table 3C (ii). This analysis shows that both AFP and GGT responses (decrease) post TARE were associated with increased survival compared with patients without these serum responses, as seen in Tables 2C (ii) and 3C (ii), although the survival differences for GGT did not quite reach significance. Thus, follow up decreased AFP levels in patients with baseline elevated levels, or follow up decreased GGT levels may be associated with improved survival. Although we did not have enough patients followed up with high CRP levels, the fact that baseline low serum CRP was associated with longer survival than for patients with high CRP levels, suggests that CRP responses at follow up might be similarly useful as were GGT and especially AFP responses.

Most current prognostication methods use one or a limited number of clinical parameters. However, this gives only a partial view of the complexities of clinical HCC parameters and their putative interactions, since prognosis is known to be affected by both tumor and by liver parameters. The network phenotyping strategy (NPS) was designed to integrate 17 standard clinical parameters into 25 prognostically-related HCC phenotypes, 1–25, with phenotype 25 having the most aggressive phenotype and worst prognosis and phenotype 1 having the least aggressive clinical characteristics and the best prognosis [23, 24, 32]. The results from this small series of TARE patients (Table 3D), show that 27.3% of patients had an improved (lower phenotype number) NPS phenotype post TARE, 40.9% of patients had a worse NPS phenotype post TARE and 31.8% had a stable phenotype. Of the 12 patients who had an improved phenotype, the 8 who started with the highest phenotype are depicted in Table 3D (ii) had a wide range of quantitative phenotype responses. Those lower (response) phenotypes reflect an improved survival [23]. This is a potentially useful clinical management tool, available at https://apkatos.github.io/webpage_nps [23, 24]. This was a retrospective study and prospective data collection with estimated changes in NPS phenotype is planned, to assess the relationship between change in NPS phenotype after TARE and survival. We examined the clinical profiles of the 4 dichotomized parameters with the highest hazard ratios, namely CRP, AFP, MTD and PVT to determine which associated parameters might be important for significant differences in survival amongst parameter pairs, shown in Table 1b. We found slightly different clinical parameter profiles (Table 4) for the 4 parameter pairs that had significant differences in survival. Thus, the profiles for the longer-survival patients (low CRP, low AFP, low MTD and absent PVT), revealed smaller tumors (MTD), lower AFP levels, lower percent of patients with PVT and less multifocality for dichotomized low CRP patients and for dichotomized low MTD patients (except MTD, by definition). Similarly for the dichotomized cohort with low percent of PVT (except PVT, by definition). Only AFP dichotomized groups were without significance for PVT or multifocality. These 2 AFP groups also showed few differences in any liver or other inflammation parameters (albumin), so the explanation for the dichotomized AFP survival differences remains unclear.

The significance of these parameter changes remain to be investigated. AFP levels are a measure of HCC aggressiveness and thus an AFP decrease represents a decrease in aggressiveness. This may also be true for levels of the HCC biomarker GGT. The reasons for an increase in ALBI or a decrease in albumin with growing HCC may reflect either the contribution of the microenvironment or its response to a growing HCC. Therefore effective HCC might be expected to result in a reversal of these. Regardless of their exact meaning, these parameter changes probably reflect the complex interactions of an HCC mass and its hepatic microenvironment. Some modern pivotal HCC treatment trials showed an increase in survival with minimal tumor size change [33].

The strengths of this study are the identification of 6 potential treatment response biomarkers, namely, ALBI, albumin, AFP, GGT, NPS and possibly CRP. Patient subsets with elevated baseline levels of all 5 of these parameters showed responses to TARE, while our follow-up data showed improved survival for patients with responses to AFP and GGT levels. Although were found patients with decreasing CRP and NPS values post TARE, larger patient numbers need follow up to determine whether these changes are associated with survival benefit. The limitations of this study are both the relatively small sample size and the loss of several patients to follow up who lived in distant places.

### Conclusions.

Several baseline parameter values predicted survival. A new survival model is described for TARE-treated HCC patients, especially for those with PVT. Several serum markers can change after TARE, including AFP, CRP, ALBI, albumin, GGT, and the newly-described NPS profile composed of 17 clinical parameters. Decreased AFP or GGT or increased albumin was associated with improved survival.

## Figures and Tables

**Table 1:** Kaplan-Meier analysis and survival comparisons by parameter.

Table 1a: Kaplan Meier analysis for all patients.		
	Survival (mo.) Mean±SE	Survival (mo.) Median±SE
All (n=116)	19.95±1.59	13.93±3.24

Table 1b: Survival comparison for each dichotomized parameter.					
	Survival (mo.) Mean±SE	Survival (mo.) Median±SE	Log-Rank p-value	HR (95% C.I.)	HR p-value
Albumin≥3.5 (n=84)	22.17±1.84	18.70±3.04	0.010	reference	
Albumin<3.5 (n=32)	13.27±2.57	5.33±0.61		1.857 (1.151–2.996)	0.011
ALBI grade 1 (n=50)	24.48±2.31	22.70±5.34	0.004	reference	
ALBI grade ≥2 (n=66)	16.01±1.95	7.40±2.96		1.978 (1.239–3.157)	0.004
PVT− (n=60)	24.07±2.15	20.87±6.79	0.002	reference	
PVT+ (n=56)	14.77±2.02	7.77±2.79		2.032 (1.288–3.206)	0.002
AFP<100 (n=57)	24.84±2.23	22.70±3.79	0.001	reference	
AFP≥100 (n=55)	14.27±2.03	7.77±2.35		2.149 (1.351–3.418)	0.001
MTD≤5 cm (n=48)	26.49±2.38	30.43±NA	<0.001	reference	
MTD>5 cm (n=68)	14.52±1.76	7.90±2.20		2.512 (1.541–4.095)	<0.001
CRP≤1 (n=49)	23.60±2.24	22.70±4.52	<0.001	reference	
CRP>1 (n=32)	11.35±2.49	5.53±0.81		2.787 (1.625–4.780)	<0.001
Hb≥13 (n=77)	20.90±1.81	18.70±3.41	0.186	reference	
Hb<13 (n=39)	17.65±2.88	6.43±1.62		1.369 (0.858–2.184)	0.188
Lymphs <1.5 (n=71)	20.87±2.08	15.33±4.01	0.492	reference	
Lymphs ≥1.5 (n=45)	18.03±2.31	13.20±4.21		1.171 (0.745–1.843)	0.493
NE≤3.5 (n=55)	24.12±2.39	20.87±4.90	0.014	reference	
NE>3.5 (n=61)	16.09±1.91	9.90±3.96		1.750 (1.111–2.756)	0.016

**Table 2:** Survival comparisons in dichotomized parameter combination groups.

		Survival (mo.) Mean±SE	Survival (mo.) Median±SE	Log-Rank p-value	HR (95% C.I.)	HR p-value
All patients	CRP≤1 & MTD≤5cm & AFP<100 (n=17)	26.30±3.63	32.03±NA	<0.001	reference	
	CRP>1 & MTD>5cm & AFP≥100 (n=13)	4.11±0.81	3.83±1.68		10.301 (3.201–33.149)	<0.001
All patients	CRP≤1 & AFP<100 (n=28)	23.09±2.85	22.70±3.67	<0.001	reference	
	CRP>1 & AFP≥100 (n=18)	6.43±1.36	4.27±0.78		4.309 (1.980–9.380)	<0.001
PVT negative	CRP≤1 & AFP<100 (n=18)	23.04±3.72	18.73±11.10	0.885	reference	
	CRP>1 & AFP≥100 (n=3)	13.12±4.64	NA		0.858 (0.108–6.806)	0.885
PVT positive	CRP≤1 & AFP<100 (n=10)	19.48±2.90	22.70±2.71	<0.001	reference	
	CRP>1 & AFP≥100 (n=15)	5.14±1.11	4.27±0.54		8.658 (2.397–31.274)	0.001
PVT positive	AFP<100 (n=24)	22.74±3.14	20.87±4.62	<0.001	reference	
	AFP≥100 (n=31)	7.64±1.29	4.97±0.59		3.852 (1.902–7.800)	<0.001
PVT positive	CRP≤1 (n=17)	18.94±2.37	22.70±2.32	<0.001	reference	
	CRP>1 (n=23)	7.75±1.86	5.33±0.85		4.333 (1.908–9.836)	<0.001

Abbreviations: as in Table 1.

**Table 3:** (A-E). ALBI, AFP, GGT and NPS changes at 3 months post TARE.

Table 3A: ALBI changes, patient # (%)* in patients with high or low AFP.		
	A (1). ALBI changes in high AFP patients n (%)	A (2). ALBI changes in low AFP patients n (%)
Decreased	18 (32.7)	19 (39.6)
Increased	7 (12.7)	7 (14.6)
Stable	30 (54.5)	22 (45.8)

Table 3B: (i). AFP changes, patient # (%)* in high baseline AFP patients.	
	n (%)
Decreased	37 (67.3)
Increased	12 (21.8)
Stable	6 (10.9)

Table 3B (ii): AFP changes in relation to survival in patients with high base line AFP levels.					
Changes in AFP levels	Survival (mo.) Mean±SE	Survival (mo.) Median±SE	Log-Rank p-value	HR (95% C.I.)	HR p-value
Decreased >50% (n=17)	34.03±3.17	NA	<0.001	reference	
Decreased ≥20% – ≤50% (n=9)	24.43±5.35	NA		2.157 (0.609–7.645)	0.223
Decreased <20% – increased (n=11)	10.17±2.58	5.53±0.95		6.855 (2.369–19.840)	0.012

Table 3C (i): GGT changes, patient # (%)* in low and high baseline AFP patients.		
	Low baseline AFP (<100)	High baseline AFP (≥100)
	GGT changes n (%)	GGT changes n (%)
Decreased	15 (34.9)	10 (32.3)
Increased	21 (48.8)	17 (54.8)
Stable	7 (16.3)	4 (12.9)

Table 3C (ii): GGT changes in relation to survival in patients with low baseline AFP values.			
Changes in GGT levels	Survival (mo.) Mean±SE	Survival (mo.) Median±SE	Log-Rank p-value
Decreased or stable (n=22)	29.72±2.98	22.7±NA	0.063
Increased (n=21)	16.36±4.49	10.9±5.85	

Table 3D: Albumin changes* in relation to survival.					
Changes in Albumin levels	(.Survival (mo Mean±SE	(.Survival (mo Median±SE	Log-Rank p-value	HR (.C.I 95%)	HR p-value
(Increased (n=13	34.76±4.04	NA	0.001>	reference	
(Stable (n=32	31.59±2.56	NA		(0.402–3.984) 1.265	0.688
(Decreased (n=22	16.38±2.74	7.77±7.84		(1.537–13.894) 4.622	0.006

Table 3E (i): NPS changes**.	
	NPS changes n (%)**
Decreased	12 (27.3)
Increased	18 (40.9)
Stable	14 (31.8)

Table 3E (ii): Changes in individual responding patients with highest baseline NPS phenotypes***.	
Baseline NPS phenotype	Response NPS phenotype
25 --->	10
23 --->	18
23 --->	14
20 --->	8
18 --->	14
17 --->	13
17 --->	5
12 --->	5

* % of patients with changes at 3 mo post TARE compared with baseline values; for AFP, this relates only to patients with baseline elevated AFP values. AFP response was a decrease ≥50%. ALBI grade decrease was a change from grade 3 to 2 or 1, or from grade 2 to 1, and conversely for an increase. Albumin decrease was a change in Child-Pugh class >3.5g/dL vs. 2.8–3.5 g/dL vs. < 2.8g/dL.

** % of patients with changes in phenotype category at 3 mo post TARE in relation to baseline values, with highest phenotype being the poorest survival category).

*** NPS phenotype number. Highest (25) has worst survival; lowest (1) has best survival

**Table 4:** Baseline parameter dichotomizations and clinical characteristics.

Table 4a: CRP baseline dichotomization and clinical characteristics as continuous variables.							
	CRP≤1 mg/dL			CRP>1 mg/dL			
	n	Median (IQR)	(Min.-Max.)	n	Median (IQR)	(Min.-Max.)	p
MTD (mm)	49	58 (52)	(12–126)	32	94 (82.5)	(25–169)	0.003
AFP	48	48.74 (654.56)	(1.26–76057)	30	1203.89 (5617.67)	(2.22–54000)	0.017
Albumin	49	4.2 (0.9)	(2.5–5.2)	32	3.65 (1.12)	(2.4–4.7)	<0.001
T.Bilirubin	49	0.9 (0.56)	(0.2–2.2)	32	0.95 (0.65)	(0.4–7.7)	0.985
GGT	49	62 (83)	(13–320)	32	157 (186.75)	(41–715)	<0.001
ALKP	49	103 (65)	(38–293)	32	148 (167.25)	(66–509)	<0.001
AST	49	35 (24.5)	(16–290)	32	68 (70.5)	(15–407)	<0.001
ALT	49	27 (19)	(10–160)	32	44 (51.25)	(10–672)	0.029
Hb	49	14.2 (3)	(7.9–17.1)	32	12.8 (1.9)	(9.7–17.5)	0.025
PLT	49	152 (112.5)	(65–486)	32	158.5 (157.75)	(30–688)	0.935
LY#	49	1.47 (0.77)	(0.42–3.15)	32	1.23 (0.7)	(0.27–12.9)	0.129
NE#	49	3.62 (1.85)	(0.91–13.93)	32	4.46 (5.07)	(2.2–12.05)	0.025

Table 4b: CRP baseline dichotomization and clinical characteristics as categorical variables.				
		CRP≤1 n (%)	CRP>1 n (%)	p
Multifocality	No	21 (42.9)	5 (15.6)	0.020
	Yes	28 (57.1)	27 (84.4)	
PVT	No	32 (65.3)	9 (28.1)	0.002
	Yes	17 (34.7)	23 (71.9)	

Table 4c: AFP baseline dichotomization and clinical characteristics as continuous variables.							
	AFP<100 IU/mL			AFP≥100 iU/mL			
	n	Median (IQR)	(Min.-Max.)	n	Median (IQR)	(Min.-Max.)	p
MTD (mm)	57	50 (57)	(12–149)	55	77 (55)	(25–165)	0.021
Albumin	57	3.8 (0.95)	(2.7–5.2)	55	4 (0.9)	(2.4–5.2)	0.958
T.Bilirubin	57	1 (0.55)	(0.4–7.7)	55	0.9 (0.7)	(0.2–2.4)	0.527
CRP	40	0.38 (1.19)	(0.30–6.93)	38	0.60 (2.45)	(0.31–43.00)	0.241
GGT	57	90 (108.5)	(13–634)	55	120 (189)	(22–1328)	0.156
ALKP	57	117 (83.5)	(38–509)	55	124 (92)	(45–528)	0.317
AST	57	43 (41.5)	(16–407)	55	50 (43)	(15–303)	0.555
ALT	57	35 (31)	(10–672)	55	30 (30)	(13–230)	0.648
Hb	57	14 (2.5)	(7.9–17.5)	55	13.8 (2.9)	(6.7–17.7)	0.988
PLT	57	120 (113)	(44–454)	55	196 (111)	(30–688)	0.002
LY#	57	1.3 (0.63)	(0.35–12.9)	55	1.43 (0.83)	(0.39–3.62)	0.162
NE#	57	3.09 (1.5)	(0.91–11.57)	55	4.53 (4.22)	(1.65–13.93)	<0.001

Table 4d: AFP baseline dichotomization and clinical characteristics as categorical variables.				
		AFP<100 IU/mL n (%)	AFP≥100 IU/mL n (%)	p
Multifocality	No	22 (38.6)	13 (23.6)	0.133
	Yes	35 (61.4)	42 (76.4)	
PVT	No	33 (57.9)	24 (43.6)	0.187
	Yes	24 (42.1)	31 (56.4)	

Table 4e: MTD baseline dichotomization and clinical characteristics as continuous variables							
	MTD≤5 cm			MTD>5 cm			
	n	Median (IQR)	(Min.-Max.)	n	Median (IQR)	(Min.-Max.)	p
AFP	46	37.32 (635.02)	(1.26–22283)	66	315.36 (3589.45)	(2.22–76057)	0.009
Albumin	48	4 (0.87)	(2.7–5.2)	68	3.8 (1)	(2.4–5.2)	0.266
T.Bilirubin	48	0.99 (0.51)	(0.4–2.8)	68	0.9 (0.62)	(0.2–7.7)	0.290
CRP	34	0.34 (0.80)	(0.31–43.00)	47	0.60 (2.42)	(0.30–20.00)	0.211
GGT	48	89.5 (107)	(13–388)	68	123 (179)	(22–1328)	0.025
ALKP	48	99 (58.25)	(38–268)	68	131 (105)	(45–528)	0.006
AST	48	37 (36.75)	(15–290)	68	51 (47.75)	(16–407)	0.018
ALT	48	29.5 (29.25)	(10–160)	68	34 (30.25)	(10–672)	0.447
Hb	48	13.6 (2.82)	(6.7–17.7)	68	13.9 (2.55)	(9–17.5)	0.541
PLT	48	119.5 (83.25)	(30–378)	68	190.5 (131.5)	(43–688)	<0.001
LY#	48	1.25 (0.87)	(0.27–3.62)	68	1.4 (0.68)	(0.39–12.9)	0.201
NE#	48	3.2 (1.95)	(0.91–8.11)	68	3.96 (3.74)	(1.43–13.93)	0.002

Table 4f: MTD baseline dichotomization and clinical characteristics as categorical variables.				
		MTD≤5cm n (%)	MTD>5cm n (%)	p
Multifocality	No	23 (47.9)	15 (22.1)	0.006
	Yes	25 (52.1)	53 (77.9)	
PVT	No	42 (87.5)	18 (26.5)	<0.001
	Yes	6 (12.5)	50 (73.5)	
Abbreviations: MTD, maximum tumor diameter; PVT, portal vein thrombosis.				

Table 4g: PVT baseline dichotomization and clinical characteristics as continuous variables.							
	PVT negative			PVT positive			
	n	Median (IQR)	(Min.-Max.)	n	Median (IQR)	(Min.-Max.)	p
MTD (mm)	60	40 (29)	(12–116)	56	94 (50)	(35–169)	<0.001
AFP	57	40.35 (765.96)	(1.26–37159)	55	182.32 (6317.51)	(2.22–76057)	0.049
Albumin	60	4 (1)	(2.7–5.2)	56	3.8 (0.9)	(2.4–4.9)	0.238
T.Bilirubin	60	1.07 (0.63)	(0.43–2.8)	56	0.89 (0.56)	(0.2–7.7)	0.031
CRP	41	0.33 (0.54)	(0.31–7.36)	40	1.37 (2.47)	(0.30–43)	0.001
GGT	60	80.5 (96)	(13–1328)	56	136.5 (194.5)	(22–715)	0.002
ALKP	60	105 (60.5)	(38–528)	56	131.5 (143)	(50–509)	0.008
AST	60	38 (39.5)	(15–380)	56	51 (47)	(16–407)	0.027
ALT	60	32.5 (30)	(10–672)	56	30 (27)	(10–434)	0.927
Hb	60	14.3 (2.2)	(6.7–17.7)	56	13.8 (2.9)	(7.9–16.4)	0.194
PLT	60	125 (94)	(30–378)	56	208 (143)	(43–688)	<0.001
LY#	60	1.38 (0.65)	(0.27–3.62)	56	1.39 (0.72)	(0.39–12.9)	0.556
NE#	60	3.39 (2)	(1.15–9.68)	56	3.96 (4.62)	(0.91–13.93)	0.012

Table 4h: PVT baseline dichotomization and clinical characteristics as categorical variables.				
		PVT negative n (%)	PVT positive n (%)	p
Multifocality	No	27 (45.0)	11 (19.6)	0.007
	Yes	33 (55.0)	45 (80.4)	

Abbreviations for Table 4: MTD, maximum tumor diameter (cm); PVT, portal vein thrombosis; Alb, albumin (g/dL); AFP, alpha-fetoprotein (IU/L); CRP, C-reactive protein (mg/dL); Hb, hemoglobin (g/dL); NE, neutrophils (x 10^9^ cells/L); Lymphs, lymphocytes (x 10^9^ cells/L); T. bilirubin, total bilirubin (mg/dL); GGT, gamma.glutamyltransferase (IU/mL); ALKP, alkaline phosphatase (IU/mL); AST, aspartate amino transferase (IU/mL); ALT, alanine amino transferase (IU/mL); PLT, platelets (x 10^9^/L).

## Abbreviations:

HCC: Hepatocellular Carcinoma
TARE: Transarterial Radioembolization
90Y: Radioactive 90Yttrium Microspheres
AFP: Alpha-Fetoprotein
MTD: Maximum Tumor Diameter
PVT: Portal Vein Invasion
CRP: C-Reactive Protein
AlB: Albumin
HB: Hemoglobin
NE: Neutrophils
Lymphs: Lymphocytes
GGT: Gamma Glutamyl Transferase
ALKP: alkaline phosphatase
AST: Aspartate Amino Transferase
ALT: Alanine Amino Transferase
PLT: Platelets
ALBI: Albumin-Bilirubin Grade
NPS: Network Phenotyping Strategy
CAT: Scan: Computed Axial Tomography Scan
